# Heat and health of occupational workers: a short summary of literature

**DOI:** 10.1093/joccuh/uiae018

**Published:** 2024-04-11

**Authors:** Sai Venkata Sarath Chandra N, Zhiwei Xu

**Affiliations:** School of Medicine and Dentistry, Parklands Drive, Southport, Gold Coast Campus, Griffith University, QLD 4222, Australia; School of Medicine and Dentistry, Parklands Drive, Southport, Gold Coast Campus, Griffith University, QLD 4222, Australia

**Keywords:** heat waves, workers, health

## Abstract

Globally, occupational workers suffer various health impacts due to extreme heat. In this short review, we examine the literature discussing health impacts of heat on occupational workers, and then discuss certain individual and institutional measures needed to address the problem. Though the available literature in the recent decade discusses health impacts of heat on workers as various heat-related illnesses, we found very few studies examining how occupational workers suffer from issues concerning cardiovascular health, neurological health, respiratory health, and mental health. In this regard, we highlight the need for more studies to examine how occupational workers exposed to extreme heat conditions suffer from fatal health issues like cardiovascular attack, brain stroke, and other ailments impacting vital organs of the body. Occupational workers across the world should be made aware of measures to protect themselves from extreme heat. Further, countries should develop occupational heat safety guidelines with statutory effect.

## Key points

- There is a need for more research to understand how extreme heat exposure impacts the risks of fatal health problems among workers.
- To protect workers, there is a need to strictly implement occupational heat safety guidelines.
- Protective measures such as mandatory outdoor work bans during hot weather enforced in some West Asian countries need to be considered.

## 1. Introduction

Occupational workers are categorized as one of the most at-risk groups threatened by heatwaves. The potential consequences of occupational heat stress on workers, workplaces, and global economies in the context of climate change are substantial.^1^ The impact of heat exposure can be particularly harsh on outdoor workers such as those engaged in agriculture, construction, mining, manufacturing sectors, and also on armed forces personnel and firefighters.^2^ Therefore, it is important to address the burden of heat on workers because global projected increases in mean temperatures and heatwaves may aggravate the risk of adverse heat health effects and exacerbate disparities.^3^ Against the backdrop of existing research on the occupational heat-health problem, our study highlights that studies of extreme heat impacts on workers must consider not just heat-related illnesses but also long-term health consequences impacting cardiovascular health, neurological health, respiratory health, and mental health. Our study also suggests certain individual and institutional reforms to protect workers from heat-related illnesses and long-term health consequences.

## 2. Materials and methods

For this Opinion summary, we used the PRISMA (Preferred Reporting Items for Systematic Reviews and Meta-Analyses) approach to systematically search literature in the PubMed database. We used “occupational heat exposure” as a key search term to identify the literature.

### 2.1. Inclusion and exclusion criteria

**Figure 1:**
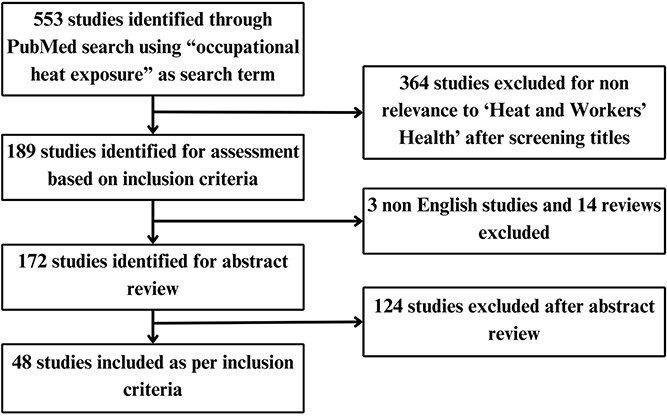
PRISMA flowchart of studies included in the summary review.

First, we included peer-reviewed publications written in English that examined the health impacts of heatwaves in occupational workers. Second, we included publications that discussed evidence-based suggestions, strategies, and measures to address the occupational heat-health problem. As we were interested in the more recent evidence, we included only papers published between January 1, 2012 and December 31, 2022. We excluded review articles and commentaries for this study. The schematic of the search strategy is shown in Figure 1. The full list of studies obtained and excluded at various stages is given in Appendix S1 along with author names and DOIs/links.

## 3. Results

We categorized the evidence as per inclusion criteria into 2 groups: (1) literature that discussed the effects of heatwaves on occupational workers and how occupational heat exposure is a problem for workers from available evidence; and (2) literature on evidence-based suggestions, strategies, and measures to address the occupational heat-health problem.

### 3.1. Heat as a health hazard for workers: summarizing global evidence

Thirty-five studies from across the world discussed heat as a health hazard for workers in various settings. Table 1 summarizes the evidence for informal occupational workers who do not have access to labour protections or social protection including construction workers, those engaged in mining activities, agricultural workers, and other industrial laborers, and also for professional workers.

**Table 1:** Evidence of workers reporting heat-health problems.

**Study**	**Region/country**	**Health illness or risk due to heat exposure**
Al-Bouwarthan et al (2020)^4^	Saudi Arabia	Impact on kidney health of construction workers
Meshi et al (2018)^5^	Tanzania	At least 1 heat-related illness among miners
Lundgren et al (2014)^6^	Chennai, India	Reporting many heat-related illnesses among agriculture and construction workers
Han et al (2021)^7^	China	Heat-related illnesses, reduced urine volume, and injuries like “falls, trips, and slips,” hitting objects, and cutting among construction workers
Calkins et al (2019)^8^	Washington State, United States	Occupational traumatic injuries among construction workers
Pogačar et al (2018)^9^	Slovenia	Gradual progress of basic heat stress symptoms to more serious forms among manufacturing workers
Tawatsupa et al (2012)^10^	Thailand	Kidney disease among workers
Crowe et al (2022)^11^	Costa Rica	Kidney injury and aggravating the risk of chronic kidney disease among agricultural workers
Venugopal et al (2019)^12^	India	Inhibiting DNA repair systems and damaging the DNA among industrial workers
Venugopal et al (2016)^13^	India	Heat-related illnesses and urogenital symptoms
Xiang et al (2016)^14^	South Australia	Heat-related illnesses
Sadiq et al (2019)^15^	Nigeria	Heavy sweating, tiredness, dizziness, and headaches among agricultural workers
Fortune et al (2013)^16^	Ontario, Canada	Increased concentration of heat-related illnesses
Wagoner et al (2020)^17^	North Mexico	Increased incidence of dehydration among agricultural workers
Wesseling et al (2016)^18^	Nicaragua	Greater risk of heat stress, dehydration, and poor renal function among sugarcane workers while construction workers and subsistence farmers also face the risk
Tang et al (2016)^19^	China	Increased blood pressure among heat-exposed steel workers due to vitamin C, potassium, and calcium losses
Sahu et al (2013)^20^	India	Lower heart rate recovery due to heat exposure indicating cardiovascular strain among the rice harvesters
Dang et al (2014)^21^	Texas, United States	Increase in postshift blood bicarbonate, blood urea nitrogen, creatinine, and urine specific gravity among the workers at an aluminum smelter
Moyce et al (2017)^22^	California, United States	Acute kidney injury and heat strain symptoms among agricultural workers
Petitti et al (2013)^23^	Maricopa County, Arizona, United States	Heat-associated deaths among those associated with construction/extraction and agriculture activity
Dally et al (2020)^24^	Southwest Guatemala	Average daily wet-bulb globe temperature >30°C increased risk of occupational injuries and dehydration among agricultural workers
Spencer et al (2022)^25^	Gambia	Headache, dizziness, nausea, and chills among pregnant women farmers
Pradhan et al (2019)^26^	Qatar	Increased cardiovascular mortality among Nepali migrant workers based in Qatar
Wang et al (2022)^27^	China	Exposure to occupational heat stress is associated with carotid atherosclerosis among male steelworkers
Habib et al (2021)^28^	Lebanon	At least 1 of heat stress-related health symptoms (fainting or dizziness, nausea, weakness, confusion, muscle cramps, hot dry skin, and chills) among bakery workers
Seng et al (2018)^29^	Singapore	Heat stress symptoms among workers in rice vermicelli manufacturing factories
Dally et al (2018)^30^	Guatemala	Evidence for the link between impaired kidney function, increased heat exposure, and agricultural worker productivity
Riccò et al (2020)^31^	Northern Italy	Increased daily rates of occupational injuries in agricultural workers from northern Italy
Hinchliffe et al (2023)^32^	Spain	No evidence for an association between heat exposure and colorectal cancer risk
Hinchliffe et al (2023)^33^	Canada, France, and Spain	No evidence for an association between heat exposure and prostate cancer risk
Raval et al (2018)^34^	Ahmedabad, India	Heavy sweating, intense thirst, dry mouth, loss of work capacity, loss of coordination, breathlessness, chest pain, loss of appetite, red eyes, headache, and stomach acidity among traffic police
Watkins et al (2021)^35^	United Kingdom	Increased cytokine levels suggestive of systemic inflammation with a risk of cardiac events among fire service instructors
Al-Otaibi et al (2022)^36^	Al-Khobar, Saudi Arabia	Increased heart rate, respiratory rate, and core body temperature and biochemical changes among bakers
Al-Otaibi et al (2022)^37^	Al-Khobar, Saudi Arabia	Abnormal hematological parameters—high hemoglobin and low platelet values among bakers
Zhou et al (2014)^38^	Jinan, China	Physiological and psychological health impacts on bus drivers
Erickson et al (2019)^39^	United States	Higher prevalence of heat-related symptoms among disaster responders

### 3.2. Interventions to address occupational heat-health threats

One study highlights the need to develop more effective prevention programs to reduce worker heat-related morbidity and mortality.^40^ Findings at a construction site in Japan indicated that heat effects on workers can be addressed by strategies that include cool drinking water availability, personal monitoring of heart rate or body water loss, providing recovery areas, voluntary breaks for workers, and providing health education for workers on heat stroke prevention strategies.^41^ Two studies discussed a “water, rest, shade” intervention to reduce the impact of heat stress on the health of sugarcane farmers.^42^^,^^43^ Two studies highlighted the importance of heat-related training programs,^7^^,^^44^ of which one called for updating existing workplace heat prevention policies, targeted and mandatory high-temperature regulations, and concerted efforts from all stakeholders.^7^ One study called for strict adherence to a heat stress management program to minimize heat stress and strain among the workers,^21^ and another highlighted preventive measures such as engineering controls and heat acclimatization programs.^29^

Some studies have highlighted how hydration can impact the health of workers. One study highlighted the need to correct poor hydration practices like irregular drinking patterns and non-plain fluid intake among outdoor workers as these are found to impact the severity of heat-related illnesses.^45^ Another study found that traditional low-cost dietary methods like consumption of buttermilk have potential to mitigate occupational heat strain by cooling the body, lowering sweat rate, improving hydration, and reducing hormonal stress.^46^ As the risk of raised blood pressure increases among heat-exposed workers due to nutrient losses, there may be a need to increase dietary intakes of vitamin C, potassium, and calcium.^19^ A study in Guatemala found that hydration could be protective to address the risk of acute kidney injury among agricultural workers exposed to heat stress.^47^

According to a study in India, female brick workers adopted a strategy of reduced walking speed to cope with physiological strain caused by extreme heat exposure.^48^ A multicountry study highlighted the need for a focus on work-rest cycles and ventilated clothing to mitigate the physiological heat strain experienced by workers.^49^ Heat monitoring at workplaces in hot tropical countries, providing shade for outdoor workers, reduction in work intensity, heat protection advice for workers in local languages, and access to clean drinking water are important to reduce occupational heat stress.^50^ One study highlighted the importance of mobile technology to provide heat safety education for farm workers,^51^ and 2 studies discussed the development of heat-health warning systems in the occupational context for the protection of workers.^52^^,^^53^

## 4. Discussion and future direction

Whereas most of the available evidence discussed the health impacts of heat exposure on workers as heat-related illnesses including headaches, dizziness, and fainting, only 5 studies^10^^,^^11^^,^^18^^,^^22^^,^^30^ discussed heat-related health disorders such as kidney disorders in workers. There is little evidence regarding the impacts of heatwaves on cardiovascular health, neurological health, respiratory health, or mental health of workers. Though there are studies that have attributed heatwaves as a major predisposing factor triggering heart, brain, lung, and mental health illnesses for the general public, we find limited evidence in the context of occupational workers. To establish extreme heat as a major risk factor for occupational workers, we need more evidence-based studies to discuss impacts on cardiological health, neurological health, respiratory health, and mental health for occupational workers. Our study suggests that research in the context of heatwaves and occupational workers must focus more on heat-related health disorders over heat-related illnesses.

**Figure 2:**
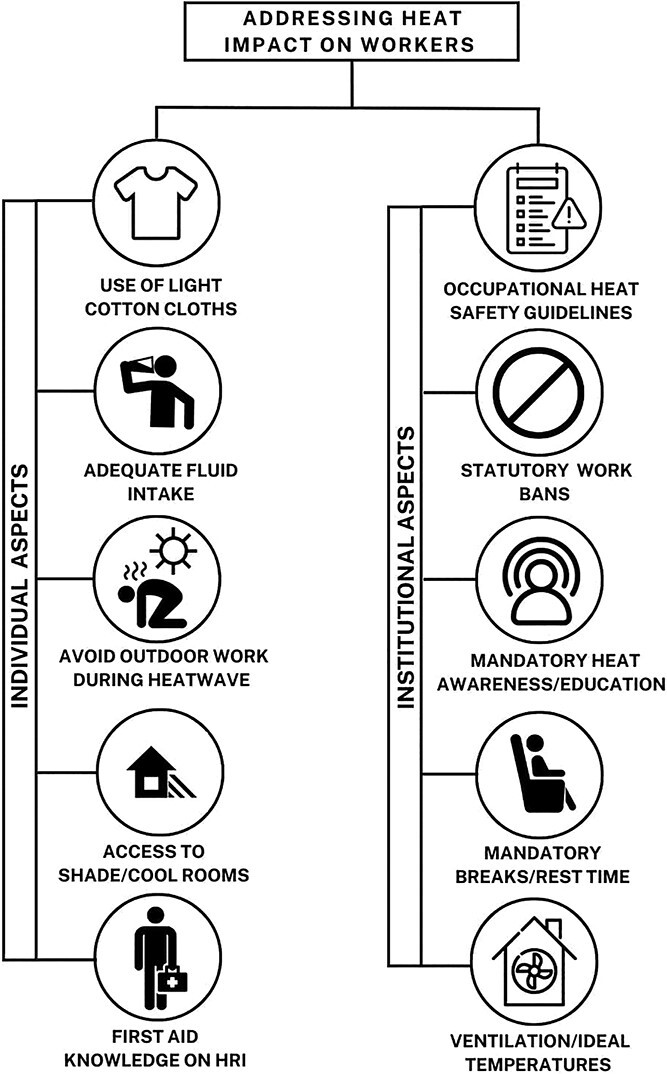
Strategies to address heat impacts on the health of workers.

Next, we highlight individual strategies to address the impact of heatwaves on workers (Figure 2). Among the individual protective strategies for workers, consumption of clean drinking water, regular hydration, accessing shade, resting whenever uncomfortable, and self-reporting of heat-related illness symptoms are predominantly emphasized. As described by a study on the benefits of traditional drinks like buttermilk that can provide a cooling effect, popularizing the consumption of local traditional drinks that have an effective hydration and cooling effect could be beneficial for workers.

As the health impacts of heatwaves on occupational workers working outdoors continue to worsen globally, there is a need for institutional measures by developing statutory and legislative provisions to address the challenge. Some countries in West Asia have incorporated mandatory work bans as a part of “workplace safety and health guidelines” when temperatures breach certain maximum thresholds. For example, Qatar’s rules on workers prohibit work in outdoor workspaces from 11:30 am to 3:00 pm, between June 15 and August 31, and regardless of the time, all work must stop if the wet-bulb globe temperature (WBGT) rises beyond 32.1°C in a particular workplace^54^; Saudi Arabia and UAE enforce a 3-month ban on working under the sun between noon and 3:00 pm,^55^^,^^56^ and Bahrain enforces a ban for 2 months.^57^ We suggest other countries could also adopt measures such as work bans during extreme heat conditions. When deaths occur among the workers, there must be reforms to update death records to highlight the cause of mortality as heat exposure.^26^

Further, occupational heat safety guidelines of all countries globally must contain provisions to provide cooling appliances to bring temperatures down at work places. There must also be mandatory checks on measures to ensure adequate ventilation and maintain ambient temperatures at work places during heatwave conditions. The guidelines also highlight the importance of “work appropriate clothing” like the use of light cotton clothing in hot working conditions. It is also important to recognize heat stroke as an industrial accident to ensure that affected workers are eligible for compensation. In the future, it is also important for countries to study occupational heat safety guidelines and legislation of countries that adopt best practices and have shown positive results in addressing the problems of occupational workers during heatwaves.

## Author contributions

N.S.V.S.C. performed the literature search and wrote the original draft. Z.X. critically reviewed the draft and provided constructive feedback to improve the manuscript.

## Supplementary data

Supplementary material is available at *Journal of Occupational Health* online.

## Funding

No funding, grants, or other support was received by either of the authors to assist with the preparation of this article.

## Conflicts of interest

The authors declare no conflicts of interest that influence the work reported in this paper.

## Data availability

The data underlying this article are available in the article and in its online supplementary material.

## Supplementary Material

Web_Material_uiae018
